# Exploratory Longitudinal Study of Ocular Structural and Visual Functional Changes in Subjects at High Genetic Risk of Developing Alzheimer’s Disease

**DOI:** 10.3390/biomedicines11072024

**Published:** 2023-07-18

**Authors:** Inés López-Cuenca, Lidia Sánchez-Puebla, Elena Salobrar-García, María Álvarez-Gutierrez, Lorena Elvira-Hurtado, Ana Barabash, Federico Ramírez-Toraño, José A. Fernández-Albarral, José A. Matamoros, Alberto Nebreda, Alejandra García-Colomo, Ana I. Ramírez, Juan J. Salazar, Pedro Gil, Fernando Maestú, José M. Ramírez, Rosa de Hoz

**Affiliations:** 1 Ramon Castroviejo Institute for Ophthalmic Research, Complutense University of Madrid, 28040 Madrid, Spain; inelopez@ucm.es (I.L.-C.); lidsan02@ucm.es (L.S.-P.); elenasalobrar@med.ucm.es (E.S.-G.); maralv25@ucm.es (M.Á.-G.); marelvir@ucm.es (L.E.-H.); joseaf08@ucm.es (J.A.F.-A.); jomatamo@ucm.es (J.A.M.); airamirez@med.ucm.es (A.I.R.); jjsalazar@med.ucm.es (J.J.S.); 2 Health Research Institute of the Hospital Clínico San Carlos (IdISSC), 28040 Madrid, Spain; ana.barabash@gmail.com (A.B.); pgil@salud.madrid.org (P.G.); fmaestuu@psi.ucm.es (F.M.); 3 Department of Immunology, Ophthalmology and ENT, Faculty of Optics and Optometry, Complutense University of Madrid, 28037 Madrid, Spain; 4 Endocrinology and Nutrition Department, Hospital Clínico Universitario San Carlos, 28040 Madrid, Spain; 5 Centre for Biomedical Research Network on Diabetes and Associated Metabolic Diseases (CIBERMED), 28029 Madrid, Spain; 6 Department of Medicine II, School of Medicine, Complutense University of Madrid, 28040 Madrid, Spain; 7 Center for Cognitive and Computational Neuroscience Laboratory of Cognitive and Computational Neurscience, Complutense University of Madrid, 28223 Pozuelo de Alarcón, Spain; federami@ucm.es (F.R.-T.); albnebre@ucm.es (A.N.); algarc23@ucm.es (A.G.-C.); 8 Department of Experimental Psychology, Cognitive Psychology and Speech & Language Therapy, Complutense University of Madrid, 28223 Pozuelo de Alarcón, Spain; 9 Memory Unit, Geriatrics Service, Hospital Clínico San Carlos, 28040 Madrid, Spain; 10 Department of Medicine, School of Medicine, Complutense University of Madrid, 28040 Madrid, Spain; 11 Biomedical Research Networking Center in Bioengineering, Biomaterials and Nanomedicine, 28029 Madrid, Spain; 12 Department of Immunology, Ophthalmology and ENT, Faculty of Medicine, Complutense University of Madrid, 28040 Madrid, Spain

**Keywords:** Alzheimer’s disease, genetic risk, familiar history, ApoE ε4, optical coherence tomography, visual acuity, contrast sensitivity

## Abstract

This study aimed to analyze the evolution of visual changes in cognitively healthy individuals at risk for Alzheimer’s disease (AD). Participants with a first-degree family history of AD (FH+) and carrying the Ε4+ allele for the ApoE gene (ApoE ε4+) underwent retinal thickness analysis using optical coherence tomography (OCT) and visual function assessments, including visual acuity (VA), contrast sensitivity (CS), color perception, perception digital tests, and visual field analysis. Structural analysis divided participants into FH+ ApoE ε4+ and FH− ApoE ε4− groups, while functional analysis further categorized them by age (40–60 years and over 60 years). Over the 27-month follow-up, the FH+ ApoE ε4+ group exhibited thickness changes in all inner retinal layers. Comparing this group to the FH− ApoE ε4− group at 27 months revealed progressing changes in the inner nuclear layer. In the FH+ ApoE ε4+ 40–60 years group, no progression of visual function changes was observed, but an increase in VA and CS was maintained at 3 and 12 cycles per degree, respectively, compared to the group without AD risk at 27 months. In conclusion, cognitively healthy individuals at risk for AD demonstrated progressive retinal structural changes over the 27-month follow-up, while functional changes remained stable.

## 1. Introduction

By 2050, dementia is expected to affect nearly 152 million people worldwide [1], with Alzheimer’s disease (AD) being the most common cause, responsible for 50–70% of all cases [2]. AD pathophysiology is known to be initiated several years before the first clinical signs appear [3]. Traditionally, studies have focused on describing the clinical stages of the disease, from mild cognitive impairment (MCI) to established disease [4]. However, there are very few studies that have addressed the changes that may occur in subjects at significant genetic risk for developing AD [5,6,7]. These very early stages would be key to understanding the early onset of AD which would be the stage at which modulating intervention would be possible [8].

Among the genetic risk factors for the development of AD are [6]: (i) presence of the ApoE ε4 allele [9], where the number of copies of ε4 influences the level of risk and is related to the age of presentation of the clinical disease [10]; and (ii) a first-degree family history of AD, which increases the probability of developing this neurodegeneration in the future by 2.9 to 6.1 times compared to subjects with no family history [11]. In this respect, descendants of individuals with AD propensity display decreased brain metabolism of cerebral areas that are affected in neurodegenerative pathology such as the posterior cingulate, precuneus, medial temporal and parietal cortex [12,13].

In addition to brain changes, given the close relationship of this structure with the retina, retinal changes observed by optical coherence tomography (OCT) have opened up new research paths. Previously, in subjects with significant genetic risk factors for the development of AD (family history and carrier of the ApoE ε4 allele), we demonstrated that thickness changes appear in different retinal layers [7], as well as in the choroidal [14] and retinal vasculature [15]. Furthermore, in these subjects, the sectors of the retina that have volume changes have been correlated with brain areas involved in AD such as the lingual gyrus, the entorhinal cortex, and the hippocampus [16].

Visual function has also been shown to be impaired in AD [17]. In fact, in cognitively healthy subjects with two genetic risk factors for the development AD (FH+ and ApoE ε4+), it has been shown that there is an increase in VA and that this correlates with a lower M100 latency and a higher power time–frequency cluster measured by magnetoencephalography (MEG) [18].

Most of the studies that analyze the retina and visual function have been performed as a descriptive observational study design and in most studies when cognitive decline already exist or when there is an AD diagnosis [19,20,21]. Nevertheless, there is still a requirement for future longitudinal trials involving individuals who are at a higher risk of developing dementia caused by AD. This type of study is of special interest to know how subjects without memory complaints and at high risk of developing AD evolve over time. Therefore, the purpose of this study is to analyze how structural and functional alterations of the visual pathway progress at 27-month follow-up in a population of cognitively healthy subjects, without neurological or ophthalmological diseases, but with significant genetic risk of developing AD (family history positive and carriers of ApoE ε4+).

## 2. Materials and Methods

### 2.1. Participants

Subjects were enrolled from the study “The cognitive and neurophysiological characteristics of participants at high genetic risk of developing dementia: a multidimensional approach” (COGDEM study) which was subsidized by the Spanish Ministry of Economy and Competitiveness with project code PSI2015-68793-C3-2-R. All participants had an age of 40–75 years; they did not have psychiatric or neurological disorders or, structural alterations on MRI. Furthermore, these subjects were free of addictions or chronic use of neuroleptic, anxiolytic, anticonvulsant, narcotic, or sedative–hypnotic sedative medications and were cognitively healthy. All subjects had undergone a complete neuropsychological screening and did not present any abnormalities.

Two cohorts were subjected to analysis. On one hand, there was a group comprising individuals lacking a familial history of sporadic Alzheimer’s disease (AD) with senile onset. These participants demonstrated the following characteristics: (i) normal cognitive function; (ii) absence of a first-degree family history of AD (FH−); (iii) non-carriers of the ApoE ε4 allele; and (iv) absence of memory recall complaints. On the other hand, the second study group consisted of individuals at a significant genetic risk of developing AD, and they were matched with the control group in terms of age and socioeconomic status. This group consisted of participants with the following attributes: (i) normal cognitive function; (ii) a first-degree family history of senile AD (FH+) (either the father or mother having AD); (iii) carrying one or more ε4 alleles for the ApoE gene (ApoE ε4+); and (iv) no memory-related complaints and no presence of vascular or other types of dementia. All participants in both cohorts obtained scores exceeding 26 on the Mini-Mental State Examination (MMSE).

Informed written consent was obtained from the participants for their inclusion in the study, and the study protocol received approval from the Local Ethics Committee (Hospital Clínico San Carlos) under the internal code 18/422-E_BS. Finally, the research adhered to the principles outlined in the Declaration of Helsinki.

**Figure 1:**
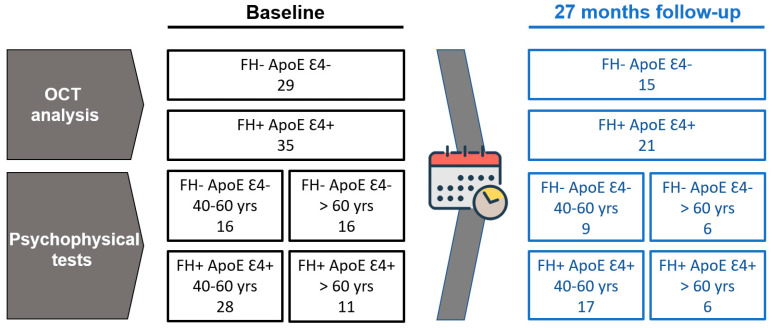
Diagram of the study groups in the baseline and 27-month follow-up examination. In black, participants included in the baseline visit, in blue, participants with follow-up visit. OCT: optical coherence tomography; FH: family history; ApoE: apolipoprotein E; yrs: years old.

An average of 27 months had elapsed between the baseline visit and the follow-up visit. At the baseline visit, OCT data from 29 participants in the FH− ApoE ε4− group and 35 subjects in the FH+ ApoE ε4+ group were analyzed. At the follow-up visit, we analyzed 15 subjects with FH− ApoE ε4− and 21 participants with FH+ ApoE ε4+ (Figure 1).

To analyze all psychophysical tests, patients were classified into two groups based on their age: a group between 40 and 60 years old, and a group over 60 years old. This classification was made taking into account the deterioration of vision that occurs from the age of 60 onwards. The groups’ distributions are detailed in Figure 1.

Patients were explored since May 2019 to September 2021.

### 2.2. Optical Coherence Tomography (OCT)

The analysis of macular region thickness was performed using OCT images obtained using the Spectralis OCT system (Heidelberg Engineering, Heidelberg, Germany), following the established OCT protocol [7].

The Heidelberg OCT utilizes segmentation software (Heidelberg, Germany, version 1.10.4.0) that enables estimation of retinal layer thickness in the macular area, including total retinal thickness. These measurements were evaluated by the same optometrist (IL-C) and manually adjusted in case of segmentation errors made by the software. The following retinal layers were examined: retinal pigment epithelium (RPE), outer nuclear layer (ONL), outer plexiform layer (OPL), inner nuclear layer (INL), inner plexiform layer (IPL), ganglion cell layer (GCL), and retinal nerve fiber layer (RNFL). The macular area analysis followed the standard of the Early Treatment Diabetic Retinopathy Study (ET-DRS). Peripapillary RNFL (pRNFL) was assessed in six sectors: nasal (N), inferonasal (IN), superonasal (SN), inferotemporal (IT), superotemporal (ST), and temporal (T). Additionally, a global mean of all peripapillary sectors was calculated. Only macular scans meeting the following quality criteria were included: an average of 16 B-scans and a minimum signal-to-noise ratio of 25. pRNFL images were required to have a minimum of 40 B-scans and a minimum signal-to-noise ratio of 20. Thickness measurements were reported in microns, based on the calibration provided by the device manufacturers.

### 2.3. Psychophysical Test

#### 2.3.1. Visual Acuity

The best monocular visual acuity (VA) was assessed using a standard clinical Snellen eye chart, following the methodology described by Salobrar-García et al. [22]. Best corrected VA was measured, and subjects began reading each row from top to bottom. VA was recorded when subjects could no longer recognize at least five out of eight letters (56.25%), which represented the highest point on the psychometric acuity function.

#### 2.3.2. Contrast Sensitivity

With the best corrected VA for far vision, we analyzed the CS using the CSV-1000E system (VectorVision, Greenville, OH, USA). Both the illumination at which the test should be performed, and the distance, followed the manufacturer’s recommendations. Four spatial frequencies were analyzed: 3, 6, 12 and 18 cycles per degree.

#### 2.3.3. Color Perception Test

In this study, color perception was assessed in individuals with the best corrected near vision using the Roth 28-Hue Color Test (Luneau technology, Paris, France) [23]. The number of errors was recorded and categorized as deuteranomalous, tritanomalous, or protanomalous (indicating deficiencies in perceiving green, blue, or red, respectively). Deuteranomaly errors were identified when the caps were misplaced between positions 42 and 85, while blue axis errors were determined based on the manufacturer’s manual, considering caps 43 to 64 in the incorrect position. The quantity of deutan and tritan errors was quantified. The administration of this test followed the procedure outlined in a previous study [24].

#### 2.3.4. Perception Digital Test

In order to evaluate visual perception impairments in patients with Alzheimer’s disease (AD), the PDT (perception digital test) has conventionally been employed [24,25]. This test comprises 15 slides, each displaying an image positioned differently in space and distorted using specific effects, such as geometric effects (mosaic) and the 24/48-frame effect implemented in the program. Consequently, the patient is required to identify the correctly oriented image, and successful completion of the test is achieved when all plates are correctly identified by the subject.

#### 2.3.5. Visual Field

Visual field testing was performed using the Humprey740i perimeter (Carl Zeiss Meditec, Dublin, CA, USA), using the SITA FAST 30-2 analysis strategy. The trial lens was calculated from the patient’s best corrected VA for distance. The test was performed in a darkened room and monocularly, occluding the non-tested eye with a patch.

The parameters analyzed in the study were: number of fixation losses, percentage of false positives and false negatives, mean deviation, deviation from the model, and visual field index.

### 2.4. Allelic Characterization

The method for detecting the APOE polymorphism has already been described in previous work [7,16]. The genotype was determined using Taqman assay technology. For this purpose, DNA was extracted from a peripheral blood sample collected in an EDTA tube using standard protocols. The C____904973_10 and C___3084793_20 assays were used to analyze the rs7412 and rs429358 SNPs, which conform the haplotype that determines the APOE isoforms. Allelic detection was performed using an Applied Biosystems 7500 Fast Real-Time PCR machine (Applied Biosystems, Foster City, CA, USA), using the appropriate assay quality controls.

### 2.5. Statistical Analysis

Statistical analysis was conducted using SPSS 27.0 software (SPSS Inc., Chicago, IL, USA). The data were reported as median (interquartile range) and mean ± standard deviation (SD). The chi-square test was employed to analyze qualitative variables between the study groups (FH− ApoE ε4− and FH+ ApoE ε4+). Within the same group, the Wilcoxon test was used to compare data between the baseline visit and the follow-up visit. The Mann–Whitney U test was utilized to compare follow-up visits between the highest and lowest risk groups. A *p*-value of <0.05 was considered statistically significant.

### 2.6. Colorimetric Representation

The color scale function of the Excel program (Microsoft, Redmond, Washington, DC, USA, version 2306) was used for the representation of changes in macular and peripapillary thickness between the baseline and follow-up visits of the study groups. The computer program directly assigns the color tone based on the normalized thickness change. A white color or a value of 1 is used when there is no difference, while a blue color with a value of −0.85 indicates thinning, and a red color with a value of 1.15 represents thickening.

## 3. Results

### 3.1. Demographic Study

During the baseline examination, OCT of 64 participants and visual function tests of 71 subjects were analyzed, at the 27-month follow-up examination there was a loss of participants due to the health situation caused by COVID-19 leading to analysis of OCT of 36 participants and visual function tests of 38 subjects.

The mean age of the FH− ApoE ε4− group at the baseline visit was 60.77 ± 7.28 and at the follow-up visit of 61.00 ± 7.56. In this study group, we found no statistically significant differences in the MMSE score (*p* = 0.480) between the baseline (28.52 ± 0.81) and follow up visits (28.60 ± 0.91).

In the FH+ ApoE ε4+ group, at the baseline visit, the mean age was 56.59 ± 6.58 and at the follow-up visit, the age was 58.63 ± 5.06. We also found no statistically significant differences in the MMSE score (*p* = 0.052) between the baseline (28.93 ± 0.53) and 27-month follow up visits (29.92 ± 0.70).

### 3.2. Longitudinal Study in the FH− ApoE ε4− Group

**Figure 2:**
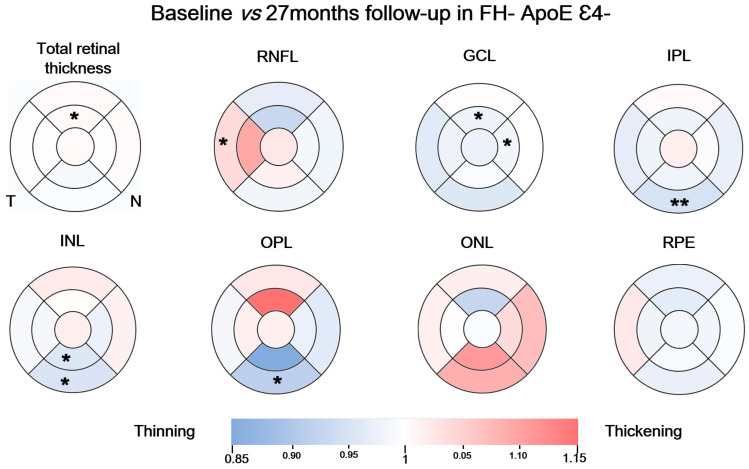
Colorimetric differences in the retinal thickness in each layer between baseline measurements and 27-month follow up in the FH− ApoE ε4− group in the macular OCT rings. In blue, thinning, in red, thickening. (RPE: retinal pigment epithelium, ONL: outer nuclear layer; OPL: outer plexiform layer; INL: inner nuclear layer; IPL: inner plexiform layer; GCL: ganglion cell layer; RNFL: retinal nerve fiber layer). * *p* < 0.05, ** *p* < 0.01. Wilcoxon test.

When we compare the macular area in the FH− ApoE ε4− group between the measurements taken at the baseline visit and the measurements taken 27 months later of the total retinal thickness, we found a statistically significant decrease in thickness in the superior sector of the inner ring (344.00 (334.50–354.00), baseline vs. 344.00 (328.25–355.75) follow-up visit) (*p* < 0.05) (Figure 2).

We also found a statistically significant increase in RNFL thickness in the temporal sector of the outer macular ring (19.00 (18.00–21.00), baseline vs. 19.50 (18.00–20.75), follow-up) (*p* < 0.05) (Figure 2).

The GCL in this group showed a statistically significant reduction in the thickness of the inner macular ring in the superior (54.00 (50.00–56.00) baseline vs. 54.00 (49.25–54.25) follow-up) (*p* < 0.05) and nasal sectors (53.00 (49.50–56.00), baseline vs. 53.00 (50.50–53.00), follow-up) (*p* < 0.05). We also found a statistically significant increase in the overall volume of this layer (1.10 (1.03–1.17), baseline vs. 1.09 (1.00–1.15), follow-up) (Figure 2).

In addition, in the IPL, a significant thickness reduction was found in the inferior sector of outer macular ring (29.00 (27.00–30.00), baseline vs. 27.00 (24.75–29.00) follow-up) (*p* < 0.01) (Figure 2) and a significant decrease in overall volume (0.93 (0.87–0.98), baseline vs. 0.93 (0.83–0.96) follow-up) (*p* < 0.05).

In the INL, the inferior sectors in both macular rings showed a significant thickness decrease (inner (43.00 (39.50–45.00), baseline vs. 40.50 (38.00–43.00) follow-up)) (outer (32.00 (30.00–34.00), baseline vs. 29.50 (28.75–34.00) follow-up)) (*p* < 0.05 in both cases) (Figure 2).

When comparing between the first measurement and at 27-month follow-up, we found a statistically significant thickness decrease in the inferior sector of the outer macular ring in the OPL (30.00 (27.00–32.50), baseline vs. 27.00 (25.75–28.50), follow-up) (*p* < 0.05) (Figure 2).

We found no statistically significant differences when comparing between baseline and follow-up in either ONL or the RPE (Figure 2).

When analyzing the pRNFL, we found a statistically significant thickness decrease in the IN sector (111.00 (102.50–132.00), baseline vs. 112.00 (99.75–125.25) follow-up) (*p* < 0.05) (Supplementary Figure S1).

### 3.3. Longitudinal Study in the FH+ ApoE ε4+ Group

**Figure 3:**
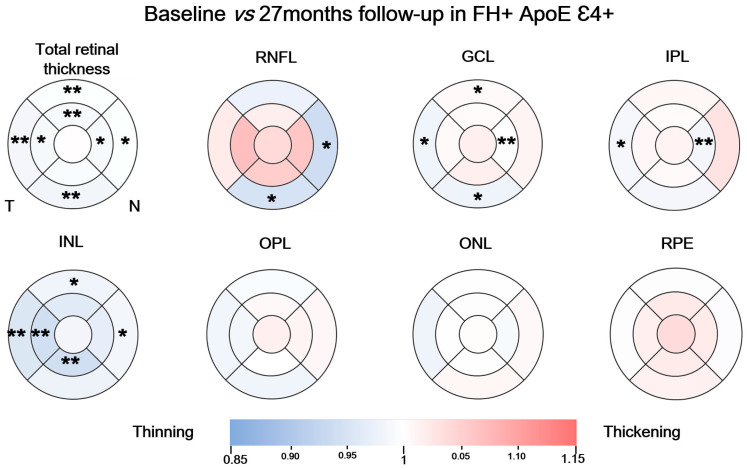
Colorimetric differences in the retinal thickness in each layer between baseline measurements and 27-month follow-up in the FH+ ApoE ε4+ group in the macular OCT rings analysis. In blue, thinning, in red, thickening. (RPE: retinal pigment epithelium, ONL: outer nuclear layer; OPL: outer plexiform layer; INL: inner nuclear layer; IPL: inner plexiform layer; GCL: ganglion cell layer; RNFL: retinal nerve fiber layer). * *p* < 0.05, ** *p* < 0.01. Wilcoxon test.

In the FH+ ApoE ε4+ group, when compared the macular thickness in the total retinal thickness measurements in the baseline with the 27-month follow-up, we observed statistically significant differences in: (i) the superior sectors of both the inner and outer rings (344.00 (339.00–352.00), baseline vs. 342 (333.5–347.25), follow-up) and (298.00 (294.00–302.00), baseline vs. 295.00 (290.00–308.00), follow-up), respectively) (*p* < 0.01 in both cases); (ii) in the nasal sector of the inner ring (345.00 (340.00–355.00), baseline vs. 342.00 (334.50–352.50) follow-up) and the outer ring (316.00 (307.00–327.00) baseline vs. (314.00 (307.00–326.00) follow-up) (*p* < 0.05 in both cases); (iii) the inferior sector of the outer ring (289.00 (282.00–297.00), baseline vs. 284.00 (277.00–291.50) follow-up) (*p* < 0.01); (iv) the temporal sector of both the inner and outer ring (329.00 (323.00–337.00), baseline vs. 284.00 (277.00–291.50) follow-up); (282.00 (277.00–290.00), baseline vs. 280.00 (267.00–285.50) follow-up), respectively (*p* < 0.01 and *p* < 0.05) (Figure 3); and in total volume (8.66 (8.48–8.83), baseline vs. 8.52 (8.31–8.77) follow-up) (*p* < 0.01) (Figure 3).

The RNFL showed a statistically significant thinning of the outer macular ring in the nasal sector (51.00 (43.00–54.00) baseline, vs. 47.00 (45.00–47.00) follow-up) (*p* < 0.05) and in the inferior sector of the outer ring (41.00 (37.00–44.00), baseline vs. 38.00 (36.50–38.00), follow-up) (*p* < 0.05) (Figure 3). We also found a statistically significant decrease in the global volume of RNFL (0.94 (0.86–1.02), baseline vs. 0.89 (0.86–0.89) follow-up) (*p* < 0.05) (Figure 3).

Furthermore, when we compared between the baseline and follow-up visits in the GCL, we observed a statistically significant thickness decrease in the nasal sector in the inner macular ring (52.00 (47.00–54.00), baseline vs. 51.00 (49.00–53.50), follow-up) (*p* < 0.01) and in the outer macular ring in the inferior and temporal sectors (33.00 (30.00–35.00), baseline vs. 33.00 (29.50–34.00), follow-up); (34.00 (32.00–37.00), baseline vs. 34.00 (31.00–37.50), follow-up), respectively (*p* < 0.05, in all cases) (Figure 3). We also found a statistically significant increase in the outer macular ring in the superior sector (34.00 (31.00–37.00), baseline vs. 34.00 (33.00–36.50), follow-up), and in the overall volume of this layer (1.08 (1.02–1.13), baseline vs. 1.07 (1.015–1.12), follow-up) (*p* < 0.01) (Figure 3).

In the IPL, we found a statistically significant thickness decrease in the nasal sector of the inner macular ring (42.00 (40.00–44.00), baseline vs. 42.00 (39.50–43.50), follow-up) (*p* < 0.01) and in the temporal sector of the outer macular ring (32.00 (30.00–34.00), baseline vs. 32.00 (29.00–33.50), follow-up) (*p* < 0.05). We also found a statistically significant reduction in the overall volume of this layer (0.90 (0.85–0.94), baseline vs. 0.90 (0.83–0.93), follow-up) (*p* < 0.05) (Figure 3).

In the INL, the thickness analysis between the baseline and follow-up shows statistically significant decrease in the inferior sector of the inner macular ring (41.50 (40.00–45.00), baseline vs. 39.00 (38.00–42.00), follow-up) (*p* < 0.01) and in the temporal sector in both macular rings (inner (38.00 (36.00–40.25), baseline vs. 36.00 (34.50–38.00), follow-up)) and (outer (32.00 (31.00–34.00), baseline vs. 32.00 (29.00–32.50), follow-up)) (*p* < 0.01 in both cases). We also found a statistically significant reduction in outer macular ring thickness in the nasal and superior sectors (34.00 (32.00–36.00), baseline vs. 33.00 (31.50–34.50), follow-up) (*p* < 0.05) and superior (31.00 (29.50–33.00) baseline, vs. 31.00 (29.50–32.00), follow-up, respectively) (*p* < 0.05, in both cases). We also found a significant reduction in the overall volume of this layer (0.96 (0.9–0.98), baseline vs. 0.93 (0.87–0.96), follow-up) (*p* < 0.05) (Figure 3).

We found no statistically significant differences when comparing between baseline and 27-month follow-up measurements in the OPL, ONL, and RPE.

In this study group, in the pRNFL we found a statistically significant thickness decrease in the temporal sector (66.00 (64.00–74.00), baseline vs. 66.00 (60.00–71.50) follow-up) (*p* < 0.05) (Supplementary Figure S1).

### 3.4. Analysis between the Study Groups at 27-Month Follow-Up

**Figure 4:**
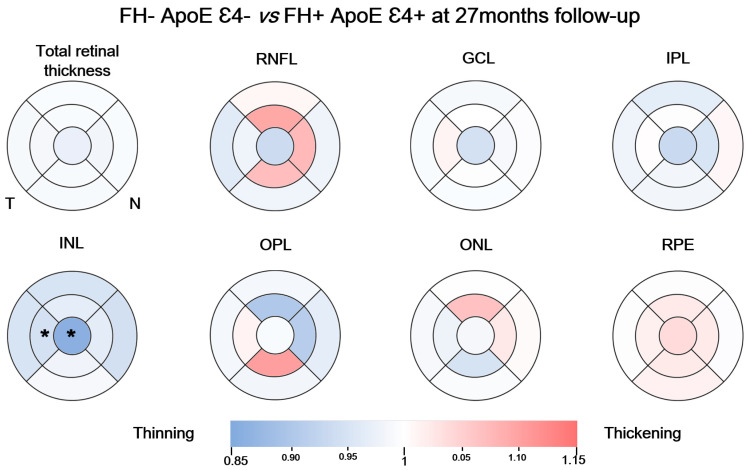
Colorimetric differences in the retinal thickness in each layer between FH− ApoE ε4− and FH+ ApoE ε4+ at 27-month follow-up. In blue, thinning, in red, thickening. (RPE: retinal pigment epithelium, ONL: outer nuclear layer; OPL: outer plexiform layer; INL: inner nuclear layer; IPL: inner plexiform layer; GCL: ganglion cell layer; RNFL: retinal nerve fiber layer). * *p* < 0.05, Mann–Whitney U test.

When we compared between the study groups, we only found statistically significant differences in INL. In this layer we observed a statistically significant reduction in thickness in the foveal sector (22.00 (18.75–23.00), FH− ApoE ɛ4− vs. 19.00 (16.00–21.00), FH+ ApoE ɛ4+) and in the temporal sector of the inner macular ring (38.00 (36.75–39.25), FH− ApoE ɛ4− vs. 36.00 (34.50–38.00), FH+ ApoE ɛ4+) (*p* < 0.05, in both cases) (Figure 4).

### 3.5. Longitudinal Psychophysical Test

#### 3.5.1. Longitudinal Study in the FH− ApoE ε4− 40–60 yrs and FH− ApoE ε4− > 60 yrs Groups

**Figure 5:**
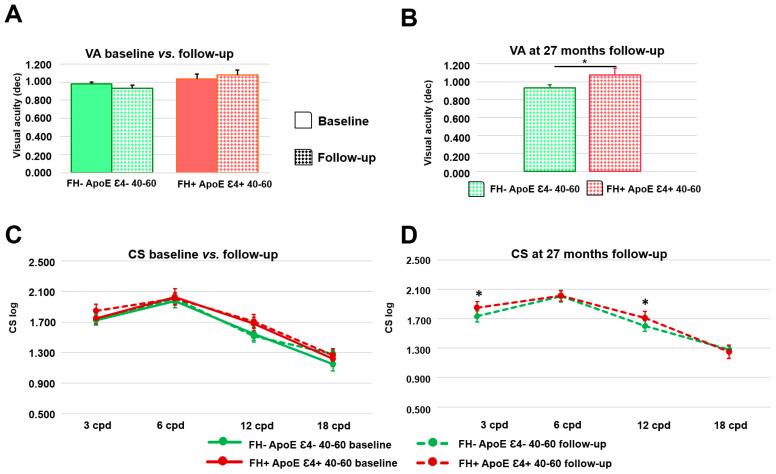
(**A**) Visual acuity between baseline and follow-up measurements in the 40–60 yrs groups. (**B**) Visual acuity at 27-month follow-up between FH− ApoE ε4− 40–60 yrs and FH+ ApoE ε4+ 40–60 yrs. (**C**) Contrast sensitivity between baseline and follow-up measurements. (**D**) Contrast sensitivity at 27-month follow-up between FH− ApoE ε4− 40–60 yrs and FH+ ApoE ε4+ 40–60 yrs. VA: visual acuity; dec: decimal, CS: contrast sensitivity; cpd: cycles per degree. (**A**,**C**) * *p* < 0.05, Wilcoxon test. (**B**,**D**) * *p* < 0.05, Mann–Whitney U test.

When we compared the results obtained at baseline and at 27-month follow-up, we found no statistically significant differences (*p* > 0.05) in the 40–60 age group in the battery of psychophysical tests analyzed (VA, CS, PDT, color perception test, and computerized perimetry) (Figure 5A and Table 1).

In the groups of participants over 60 years of age, we found no statistically significant differences in the psychophysical test analyzed (*p* > 0.05) (Supplementary Table S1).

#### 3.5.2. Longitudinal Study in the FH− ApoE Ɛ4− 40–60 yrs and FH− ApoE Ɛ4− > 60 yrs Groups

We also found no statistically significant differences between baseline and follow-up in the psychophysical tests in any of the age groups of the FH+ ApoE Ɛ4+ participants (Figure 5A,C, Table 1 and Supplementary Table S1).

#### 3.5.3. Analysis of Psychophysical Test between the Study Groups at 27-Month Follow-Up

When we compared the measurements obtained at 27-month follow-up, we found that the FH+ ApoE ε4+ 40–60 years group (1.077 ± 0.148) shows a statistically significant increase in VA compared to the FH− ApoE ε4− 40–60 yrs group (0.933 ± 0.070) (*p* < 0.05) (Figure 5B and Table 1).

Additionally, at 27 months we found statistically higher contrast sensitivity in the FH+ ApoE ε4+ 40–60 yrs group at the spatial frequencies of 3 and 12 cpd compared to the FH− ApoE ε4− 40–60 yrs group ((1.731 ± 0.148 FH− ApoE ε4− 40–60 yrs vs. 1.845 ± 0.176 FH+ ApoE ε4+ 40–60 yrs) (*p* < 0.05) and (1.511 ± 0.142, FH− ApoE ε4− 40–60 yrs vs. 1.708 ± 0.181 FH+ ApoE ε4+ 40–60 yrs) (*p* < 0.01), respectively) (Figure 5D and Table 1).

## 4. Discussion

Few longitudinal studies have been performed in participants with significant genetic risk for the development AD, and many of them consider preclinical stages of the disease when there are already memory complaints, MCI, or even positive biomarkers such as Aβ+ or pTau+ in (cerebrospinal fluid) CSF and PET. Moreover, in some cases, participants are included over time, so they cannot be said to be purely longitudinal.

Although sporadic AD is a multifactorial disease and there are numerous known and unknown genes that contribute to the increased risk of development, the presence of the ε4 allele for the ApoE gene is currently recognized as the most important [26]. Moreover, despite being cognitively healthy, carriers of this allele (ε4) already show a higher burden of Aβ than carriers of the other isoforms (ApoE Ɛ2 and Ɛ3) [27]. Therefore, characterizing this population at high genetic risk is important in order to understand the evolution of these subjects over time.

On the other hand, the inclusion of control participants is really complex because it is difficult to create a sample of controls with no risk variables if we consider that as we have mentioned above, AD is a multifactorial disease of which we do not know many factors or their interactions yet [28], therefore we have delimited participants to an age-matched population as control, with no family history of AD or vascular dementia and without any ε4 allele for the ApoE gene.

On the other hand, despite the health situation caused by COVID-19 and the loss of patients between the baseline visit and the 27-month follow-up visit, we monitored 15 subjects without significant genetic risk factors for AD and 21 subjects at risk for AD for longitudinal study.

In our study, in which we performed both structural and functional analysis, when comparing between the baseline visit and the follow-up visit at 27 months, more sectors with statistically significant retinal macular thickness changes were found in the FH+ ApoE ɛ4+ group compared with those in the FH− ApoE ɛ4− group. These changes were mostly decreases in thickness, although there were also increases, possibly due to compensatory mechanisms [29]. However, in participants who did not have significant genetic risk for AD, the changes found were increases and decreases in thickness with no statistical significance. These alterations could be due to a normal retinal aging process [30,31]. 

When we compared the FH− ApoE ɛ4− and the FH+ ApoE ɛ4+ groups at the follow-up visit, statistically significant decreases appeared in the INL. These significant thinnings occur in the foveal sector and in the temporal sector of the inner macular ring. At the first visit the changes were mostly in the IPL, which contains the synaptic connections between amacrine, bipolar, and ganglion cells [7]. This could be due to neuroinflammatory processes in which microglia perform stripping phenomena (disconnection of abnormal synapses), producing thinning in this layer. In the follow-up visit, significant thinning was found in the INL, possibly due to the death of some of these cells that began to disconnect 27 months ago [31,32,33]. Furthermore, it is noteworthy that these changes, although significant, do not become pathological, as these participants are cognitively healthy and we do not know if they will eventually develop AD.

In other longitudinal studies in subjects with Aβ+ markers, such as van de Kreeke et al., at 22-month follow-up study, they observed a thickness decrease in all retinal layers except mRNFL, but no change in retinal thickness between Aβ+ and Aβ− participants at the beginning of the study [34]. One possible explanation is the relatively brief follow-up period, and given that preclinical phases can last up to 20 years, it is possible that even if there is an increase in neurodegeneration, it is so subtle as to be difficult to detect [35].

In other study with a 27-month follow-up, the Aβ+ subjects did show a decrease in the volume of the IPL, ONL, and mRNFL, and an increase in the volume of the OPL. However, the participants of this study, in addition to having a family history of the disease, already had subjective memory complaints, and these subjects were in more advanced stages of the disease [21]. A further study also found structural changes in the retina of subjects with subjective cognitive decline and Aβ+ and that this macular thickening of the nasal region was maintained at 24 months. They also found a thinning of the RNFL in Aβ+, but without reaching statistical significance when compared to Aβ− [36]; these findings being compatible with some of the changes found in our study. A correlation has also been found between worse subjective cognitive impairment scores and a reduction in RNFL volume in a 27-month longitudinal study among subjects with a first-degree family history of AD, carriers of any ɛ4 allele for ApoE, and subjective cognitive impairment [37].

Moreover, a more recent investigation revealed that individuals carrying the ApoE ε4+ allele exhibited greater thinning in the macular central subfield thickness compared to the ApoE ε4− group. However, no statistically significant differences were observed in the percentage change rate of central macular thickness, GCL-IPL complex thickness, and RNFL thickness at the 24-month follow-up when comparing between these groups. In this study, an additional analysis was conducted to compare subjects in the ApoE ε4+ risk group who also had a family history of dementia with those in the ε4− group who did not have such a family history. No significant differences were found in any OCT parameter, both at the baseline assessment and at the 2-year follow-up. Nevertheless, the ApoE ε4+ group and subjects with a family history demonstrated a higher rate of decrease in GC-IPL thickness compared to the ApoE ε4− group without a family history [5]. However, when analyzing the demographics of the study groups, we can see that there are different races within the study groups who are subjects with an average age of over 65 years. On the other hand, the study of individual layers of the retina provides more information than the analysis of complexes. For example, when analyzing the GCL-IPL complex, information is probably lost because the ganglion cell layer has other cells such as displaced amacrine cells that can be mistaken for ganglion cells [38,39,40].

In another study that has looked at retinal changes associated with ApoE genotype and AD, the study patients had MCI and average ages of over 70 years. In addition, in this study the subjects in the control group were patients who were waiting to undergo cataract surgery. This may cause the OCT images to be of poor quality and they should be excluded from the study [41].

Changes in retinal layer thicknesses have also been detected in cognitively healthy subjects who carry the E280A presenilin 1 mutation (PSEN1). Significant thinning was found in the ONL, OPL, and INL layers [42]. The authors hypothesize that the cell death and consequent thinning of these layers may be due to loss of PSEN1 proteolytic function [43].

In our study, changes also occurred in the peripapillary area over 27 months, but when we performed a comparison at the follow-up visit between the study groups (FH− ApoE ɛ4− and FH+ ApoE ɛ4+) we found no statistically significant differences. This again highlights that during AD, changes occur early in the macular area and that changes in the peripapillary area are a sign of disease progression to more advanced stages [44,45].

Longitudinal studies have also been carried out to understand the evolution of brain changes in subjects at significant risk for AD. Shi et al., in healthy subjects, showed a correlation between the measurement of RNFL in the superior and inferior sectors and a decrease in the volume of the cingulate cortex and hippocampus, as well as the decline in episodic memory at 12 months [46]. Additionally, in a previous work, we found that in subjects with significant genetic risk factors for the development of AD there is a correlation between retinal areas where changes occur and brain areas closely related to AD such as the lingual gyrus, the hippocampus, and the entorhinal cortex [16]. Other authors have conducted longitudinal studies that also analyzed both functional brain changes and Aβ accumulation in cognitively healthy subjects and classify them by ApoE status. They found a relationship between ApoE ε4 and higher rates of change in Aβ accumulation in cognitively healthy middle-aged subjects, and therefore this accumulation changes regionally with age, being earlier in the precuneus and later in the visual cortex. These subjects show earlier and faster accumulations and higher rates of volume alterations in the structures of the medial temporal lobe. However, in these middle-aged subjects carrying ApoE ε4+, they did not perform any ophthalmological studies [47]. It has been described that these cerebral structural alterations in preclinical AD follow a biphasic pattern, first developing a thickening of the cortex due to an inflammatory process generated by the sum of the pathogenic effects between Tau and Aβ, followed by a second phase of atrophy where the effect of Tau predominates [48].

To the best of our knowledge, this is one of the first studies to analyze the visual function in subjects who are at high genetic risk for developing AD. In the present work, when we compared the follow-up visit values between the higher and lower risk group, we did find that the FH+ ApoE ɛ4+ 40–60 yrs group had statistically (*p* < 0.05) higher VA and CS at the spatial frequency of 3 and 12 cpd than the FH− ApoE ɛ4− 40–60 yrs group. These findings were already observed the first time they were evaluated, so functional changes remain stable at 27 months. The increase in VA and CS in subjects at high genetic risk for the development of AD is contrary to the findings that pointed to a progressive worsening of these visual abilities in the clinical phases [45]. This is consistent with the fact that in stage 0 disease, the pattern of atrophy mainly affects the frontal, parietal, and temporal areas with slight preservation of the primary sensory and visual cortexes [48,49,50]. In addition, it is known that early accumulation of Aβ produces alterations in the function of inhibitory neurons, which could be related to an alteration of the dopaminergic system [51,52]. It is possible that in individuals with significant genetic risk factors for the development of AD, Aβ oligomers affect the dopaminergic modulatory activity of the retina causing early hyperexcitability leading to an initial increase in VA and CS [53]. In addition, different ApoE alleles are known to confer different risks in ocular diseases leading to vision loss. The ɛ2 and ɛ3 alleles are known to predispose to an increased risk of age-related macular degeneration and the ɛ4 allele has also been associated with a decreased risk of developing glaucoma [54]. At the time the participants included in this study were analyzed, all were ophthalmologically healthy and those with ocular pathology were excluded from the study [55,56]. Among the possible limitations of our study is the fact that we have not adjusted the statistics for multiple comparisons. This is because it is a novel longitudinal exploratory study in a highly characterized study population, so our findings could be the starting point for future research in the identification of parameters of great interest in the study of high-risk populations. Another limitation of our work is the loss of participants in the longitudinal study due to the health situation caused by the COVID-19 pandemic. In addition, in the baseline visit the number of participants who underwent the psychophysical tests is higher than the number of participants who undertook the OCT. This was due to the fact that in three participants with FH− ApoE ε4− and in four participants with FH+ ApoE ε4+ OCT, images could not be included for analysis. Images with artefacts due to motion or flicker were eliminated; although artefacts are common in OCT images, they could interfere with the quantitative data.

Finally, the longitudinal study of these participants was performed at the 27-month follow-up to determine whether the changes found in the baseline visit remained stable or evolved over time. It should be considered that these subjects are healthy and that we do not know whether or not they will develop the disease in the future, so 27 months is a short follow-up time, but at least it gives us an approximation of what is happening both functionally and structurally.

## 5. Conclusions

In conclusion, in cognitively healthy subjects, but with significant genetic risk factors for the development of AD, retinal structural changes are observed that progress at 27-month follow-up, but the functional changes detected at the baseline visit remain stable, probably because of the maintenance of the hyperexcitability situation that causes the accumulation of Aβ and impairs the functioning of the dopaminergic system.

## Figures and Tables

**Table 1:** Psychophysical test measures in subjects aged 40–60 years.

		FH− ApoE ɛ4− 40–60 yrs			FH+ ApoE ɛ4+ 40–60 yrs			*p*-Value at 27 Months FH− ApoE ɛ4− 40–60 yrs vs. FH+ ApoE ɛ4− 40–60 yrs
		Baseline	Follow-Up	*p*-Value	Baseline	Follow-Up	*p*-Value	
**Visual acuity**		0.981 ± 0.040	0.933 ± 0.070	0.102	1.036 ± 0.095	1.077 ± 0.148	0.285	0.014 *
**Contrast sensitivity**	**3 cpd**	1.724 ± 0.121	1.731 ± 0.148	0.131	1.749 ± 0.148	1.845 ± 0.176	0.100	0.047 *
	**6 cpd**	1.972 ± 0.171	2.007 ± 0.158	0.943	2.028 ± 0.213	2.008 ± 0.139	0.823	0.932
	**12 cpd**	1.544 ± 0.170	1.511 ± 0.142	0.311	1.675 ± 0.181	1.708 ± 0.181	0.259	0.008 **
	**18 cpd**	1.144 ± 0.164	1.283 ± 0.100	0.119	1.219 ± 0.197	1.261 ± 0.176	0.059	0.777
**Color perception**	**Total errors**	1.500 (0.000–6.000)	2.000 (0.000–6.000)	0.127	3.000 (0.000–3.000)	2.000 (0.000–4.500)	0.304	0.710
	**Tritan errors**	1.000 (0.000–2.750)	0.000 (0.000–3.000)	0.303	0.000 (0.000–3.000)	0.000 (0.000–1.500)	0.263	0.620
	**Deutan errors**	1.000 (0.000–2.750)	0.000 (0.000–3.000)	0.347	0.000 (0.000–3.000)	0.000 (0.000–1.500)	0.205	0.853
**PDT**		0.000 (0.000–1.000)	0.000 (0.000–0.000)	0.157	0.000 (0.000–0.000)	0.000 (0.000–0.500)	0.157	0.454
**Visual Field**	**Fixation losses**	0.500 (0.000–1.000)	1.000 (0.000–1.000)	0.408	0.000 (0.000–1.000)	0.000 (0.000–1.000)	0.890	0.311
	**False positives %**	1.000 (0.000–5.000)	2.000 (0.000–2.500)	0.414	3.000 (0.000–5.750)	2.000 (0.000–6.500)	0.552	0.460
	**%False negatives**	0.000 (0.000–1.000)	0.000 (0.000–3.500)	0.273	0.000 (0.000–4.000)	1.000 (0.000–3.000)	0.959	0.954
	**VFI (%)**	99.500 (99.000–100.000)	99.000 (98.500–100.000)	0.083	100.000 (99.000–100.000)	99.000 (97.000–100.000)	0.305	0.735
	**Mean deviation (MD)**	−0.885 (−1.868–2.950)	−0.420 (−1.695–0.115)	0.484	−0.650 (−1.675–0.013)	−0.560 (−2.155–0.165)	0.999	0.936
	**Pattern standard deviation (PSD)**	1.635 (1.410–1.833)	1.640 (1.445–2.085)	0.575	1.535 (1.318–1.848)	1.660 (1.4650–2.420)	0.064	0.500

Median (interquartile range); * *p* < 0.05; ** *p* < 0.01 Wilcoxon test and Mann–Whitney U test. FH; family history; ApoE: apolipoprotein E; cpd: cycles per degree; PDT: perception digital test; VFI: visual field index.

## Data Availability

The data supporting the findings of this study are available from the corresponding author upon request.

## Supplementary Materials

The following supporting information can be downloaded at: https://www.mdpi.com/article/10.3390/biomedicines11072024/s1, Figure S1: Colorimetric differences in the peripapillary retinal nerve fiber layer thickness between baseline measurements and 27-month follow-up in the FH− ApoE ε4− and FH+ ApoE ε4+ groups and between groups at the 27-month follow-up; Table S1: Measurements of psychophysical tests in subjects over 60 years of age.

## References

[1] WHO (2021). Dementia. https://www.who.int/news-room/fact-sheets/detail/dementia.

[2] Vieira R.T., Caixeta L., Machado S., Silva A.C., Nardi A.E., Arias-Carrión O., Carta M.G. (2013). Epidemiology of early-onset dementia: A review of the literature. Clin. Pract. Epidemiol. Ment. Health CP EMH.

[3] Sperling R.A., Aisen P.S., Beckett L.A., Bennett D.A., Craft S., Fagan A.M., Iwatsubo T., Jack C.R., Kaye J., Montine T.J. (2011). Toward defining the preclinical stages of Alzheimer’s disease: Recommendations from the National Institute on Aging-Alzheimer’s Association workgroups on diagnostic guidelines for Alzheimer’s disease. Alzheimer’s Dement..

[4] Alzheimer’s association (2020). 2020 Alzheimer’s disease facts and figures. Alzheimer’s Dement..

[5] Ma J.P., Robbins C.B., Lee J.M., Soundararajan S., Stinnett S.S., Agrawal R., Plassman B.L., Lad E.M., Whitson H., Grewal D.S. (2022). Longitudinal Analysis of the Retina and Choroid in Cognitively Normal Individuals at Higher Genetic Risk of Alzheimer Disease. Ophthalmol. Retin..

[6] Ramos A.A., Galiano-Castillo N., Machado L. (2022). Cognitive Functioning of Unaffected First-degree Relatives of Individuals With Late-onset Alzheimer’s Disease: A Systematic Literature Review and Meta-analysis. Neuropsychol. Rev..

[7] López-Cuenca I., de Hoz R., Salobrar-García E., Elvira-Hurtado L., Rojas P., Fernández-Albarral J.A., Barabash A., Salazar J.J., Ramírez A.I., Ramírez J.M. (2020). Macular Thickness Decrease in Asymptomatic Subjects at High Genetic Risk of Developing Alzheimer’s Disease: An OCT Study. J. Clin. Med..

[8] Aschenbrenner A.J., Balota D.A., Gordon B.A., Ratcliff R., Morris J.C. (2016). A diffusion model analysis of episodic recognition in preclinical individuals with a family history for Alzheimer’s disease: The adult children study. Neuropsychology.

[9] Yang A., Kantor B., Chiba-Falek O. (2021). *APOE*: The New Frontier in the Development of a Therapeutic Target towards Precision Medicine in Late-Onset Alzheimer’s. Int. J. Mol. Sci..

[10] Farrer L.A., Cupples L.A., Haines J.L., Hyman B., Kukull W.A., Mayeux R., Myers R.H., Pericak-Vance M.A., Risch N., Van Duijn C.M. (1997). Effects of Age, Sex, and Ethnicity on the Association Between Apolipoprotein E Genotype and Alzheimer Disease: A Meta-analysis. JAMA.

[11] Scarabino D., Gambina G., Broggio E., Pelliccia F., Corbo R.M. (2015). Influence of family history of dementia in the development and progression of late-onset Alzheimer’s disease. Am. J. Med. Genet. Part B Neuropsychiatr. Genet..

[12] Donix M., Burggren A.C., Suthana N.A., Siddarth P., Ekstrom A.D., Krupa A.K., Jones M., Martin-Harris L., Ercoli L.M., Miller K.J. (2010). Family History of Alzheimer’s Disease and Hippocampal Structure in Healthy People. Am. J. Psychiatry.

[13] Mosconi L., Murray J., Tsui W.H., Li Y., Spector N., Goldowsky A., Williams S., Osorio R., McHugh P., Glodzik L. (2014). Brain imaging of cognitively normal individuals with 2 parents affected by late-onset AD. Neurology.

[14] López-Cuenca I., de Hoz R., Alcántara-Rey C., Salobrar-García E., Elvira-Hurtado L., Fernández-Albarral J.A., Barabash A., Ramírez-Toraño F., de Frutos-Lucas J., Salazar J.J. (2021). Foveal Avascular Zone and Choroidal Thickness Are Decreased in Subjects with Hard Drusen and without High Genetic Risk of Developing Alzheimer’s Disease. Biomedicines.

[15] López-Cuenca I., Salobrar-García E., Sánchez-Puebla L., Espejel E., del Arco L.G., Rojas P., Elvira-Hurtado L., Fernández-Albarral J.A., Ramírez-Toraño F., Barabash A. (2022). Retinal Vascular Study Using OCTA in Subjects at High Genetic Risk of Developing Alzheimer’s Disease and Cardiovascular Risk Factors. J. Clin. Med..

[16] López-Cuenca I., Marcos-Dolado A., Yus-Fuertes M., Salobrar-García E., Elvira-Hurtado L., Fernández-Albarral J.A., Salazar J.J., Ramírez A.I., Sánchez-Puebla L., Fuentes-Ferrer M.E. (2022). The relationship between retinal layers and brain areas in asymptomatic first-degree relatives of sporadic forms of Alzheimer’s disease: An exploratory analysis. Alzheimer’s Res. Ther..

[17] Tzekov R., Mullan M. (2014). Vision function abnormalities in Alzheimer disease. Surv. Ophthalmol..

[18] López-Cuenca I., Nebreda A., García-Colomo A., Salobrar-García E., de Frutos-Lucas J., Bruña R., Ramírez A.I., Ramirez-Toraño F., Salazar J.J., Barabash A. (2023). Early visual alterations in individuals at-risk of Alzheimer’s disease: A multidisciplinary approach. Alzheimer’s Res. Ther..

[19] Golzan S., Goozee K., Georgevsky D., Avolio A., Chatterjee P., Shen K., Gupta V., Chung R., Savage G., Orr C.F. (2017). Retinal vascular and structural changes are associated with amyloid burden in the elderly: Ophthalmic biomarkers of preclinical Alzheimer’s disease. Alzheimer’s Res. Ther..

[20] Van De Kreeke J.A., Nguyen H., Haan J.D., Konijnenberg E., Tomassen J., Braber A.D., Kate M.T., Collij L., Yaqub M., Van Berckel B. (2019). Retinal layer thickness in preclinical Alzheimer’s disease. Acta Ophthalmol..

[21] Santos C.Y., Johnson L.N., Sinoff S.E., Festa E., Heindel W.C., Snyder P.J. (2018). Change in retinal structural anatomy during the preclinical stage of Alzheimer’s disease. Alzheimer’s Dementia: Diagn. Assess. Dis. Monit..

[22] Salobrar-Garcia E., Hoyas I., Leal M., de Hoz R., Rojas B., Ramirez A.I., Salazar J.J., Yubero R., Gil P., Triviño A. (2015). Analysis of Retinal Peripapillary Segmentation in Early Alzheimer’s Disease Patients. BioMed. Res. Int..

[23] Roth A. (1966). Test-28 hue de Roth selon Farnsworth-Munsell (Manual).

[24] Salobrar-García E., de Hoz R., Ramírez A.I., López-Cuenca I., Rojas P., Vazirani R., Amarante C., Yubero R., Gil P., Pinazo-Durán M.D. (2019). Changes in visual function and retinal structure in the progression of Alzheimer’s disease. PLoS ONE.

[25] Rami L., Serradell M., Bosch B., Villar A., Molinuevo J.L. (2007). Perception Digital Test (PDT) for the assessment of incipient visual disorder in initial Alzheimer’s disease. Neurologia.

[26] Lozupone M., Panza F. (2024). Impact of apolipoprotein E isoforms on sporadic Alzheimer’s disease: Beyond the role of amyloid beta. Neural Regen. Res..

[27] Salvadó G., Grothe M.J., Groot C., Moscoso A., Schöll M., Gispert J.D., Ossenkoppele R., Initiative F.T.A.D.N. (2021). Differential associations of APOE-ε2 and APOE-ε4 alleles with PET-measured amyloid-β and tau deposition in older individuals without dementia. Eur. J. Nucl. Med..

[28] Papassotiropoulos A., Fountoulakis M., Dunckley T., Stephan D.A., Reiman E.M. (2006). Genetics, transcriptomics and proteomics of Alzheimer’s disease. J. Clin. Psychiatry.

[29] Jáñez-Escalada L., Jáñez-García L., Salobrar-García E., Santos-Mayo A., De Hoz R., Yubero R., Gil P., Ramírez J.M. (2019). Spatial analysis of thickness changes in ten retinal layers of Alzheimer’s disease patients based on optical coherence tomography. Sci. Rep..

[30] Alamouti B., Funk J. (2003). Retinal thickness decreases with age: An OCT study. Br. J. Ophthalmol..

[31] Ramírez A.I., Fernández-Albarral J.A., de Hoz R., López-Cuenca I., Salobrar-García E., Rojas P., Valiente-Soriano F.J., Avilés-Trigueros M., Villegas-Pérez M.P., Vidal-Sanz M. (2020). Microglial changes in the early aging stage in a healthy retina and an experimental glaucoma model. Prog. Brain Res..

[32] Trapp B.D., Wujek J.R., Criste G.A., Jalabi W., Yin X., Kidd G.J., Stohlman S., Ransohoff R. (2007). Evidence for synaptic stripping by cortical microglia. Glia.

[33] De Hoz R., Gallego B.I., Ramírez A.I., Rojas B., Salazar J.J., Valiente-Soriano F.J., Avilés-Trigueros M., Villegas-Perez M.P., Vidal-Sanz M., Triviño A. (2013). Rod-Like Microglia Are Restricted to Eyes with Laser-Induced Ocular Hypertension but Absent from the Microglial Changes in the Contralateral Untreated Eye. PLoS ONE.

[34] van de Kreeke J.A., Nguyen H.T., Konijnenberg E., Tomassen J., Braber A.D., Kate M.T., Yaqub M., van Berckel B., Lammertsma A.A., Boomsma D.I. (2020). Longitudinal retinal layer changes in preclinical Alzheimer’s disease. Acta Ophthalmol..

[35] Jansen W.J., Ossenkoppele R., Knol D.L., Tijms B.M., Scheltens P., Verhey F.R., Visser P.J., Aalten P., Aarsland D., Alcolea D. (2017). Faculty Opinions recommendation of Prevalence of cerebral amyloid pathology in persons without dementia: A meta-analysis. JAMA.

[36] Marquié M., Valero S., Castilla-Marti M., Martínez J., Rodríguez-Gómez O., Sanabria Á., Tartari J.P., Monté-Rubio G.C., Sotolongo-Grau O., on behalf of the FACEHBI Study Group (2020). Association between retinal thickness and β-amyloid brain accumulation in individuals with subjective cognitive decline: Fundació ACE Healthy Brain Initiative. Alzheimer’s Res. Ther..

[37] Cheng D.L., Thompson L., Snyder P.J. (2019). A Potential Association Between Retinal Changes, Subjective Memory Impairment, and Anxiety in Older Adults at Risk for Alzheimer’s Disease: A 27-Month Pilot Study. Front. Aging Neurosci..

[38] Nadal-Nicolás F.M., Salinas-Navarro M., Jiménez-López M., Sobrado-Calvo P., Villegas-Pérez M.P., Vidal-Sanz M., Agudo-Barriuso M. (2014). Displaced retinal ganglion cells in albino and pigmented rats. Front. Neuroanat..

[39] Hannibal J., Christiansen A.T., Heegaard S., Fahrenkrug J., Kiilgaard J.F. (2017). Melanopsin expressing human retinal ganglion cells: Subtypes, distribution, and intraretinal connectivity. J. Comp. Neurol..

[40] Sjöstrand J., Popovic Z., Conradi N., Marshall J. (1999). Morphometric study of the displacement of retinal ganglion cells subserving cones within the human fovea. Graefe’s Arch. Clin. Exp. Ophthalmol..

[41] Shin J.Y., Choi E.Y., Kim M., Lee H.K., Byeon S.H. (2021). Changes in retinal microvasculature and retinal layer thickness in association with apolipoprotein E genotype in Alzheimer’s disease. Sci. Rep..

[42] Armstrong G.W., Kim L.A., Vingopoulos F., Park J.Y., Garg I., Kasetty M., Silverman R.F., Zeng R., Douglas V.P., Lopera F. (2021). Retinal Imaging Findings in Carriers With *PSEN1*-Associated Early-Onset Familial Alzheimer Disease Before Onset of Cognitive Symptoms. JAMA Ophthalmol..

[43] Gupta V.B., Chitranshi N., den Haan J., Mirzaei M., You Y., Lim J.K., Basavarajappa D., Godinez A., Di Angelantonio S., Sachdev P. (2021). Retinal changes in Alzheimer’s disease—Integrated prospects of imaging, functional and molecular advances. Prog. Retin. Eye Res..

[44] Garcia-Martin E.S., Rojas B., Ramirez A.I., de Hoz R., Salazar J.J., Yubero R., Gil P., Triviño A., Ramirez J.M. (2014). Macular Thickness as a Potential Biomarker of Mild Alzheimer’s Disease. Ophthalmology.

[45] Salobrar-Garcia E., de Hoz R., Rojas B., Ramirez A.I., Salazar J.J., Yubero R., Gil P., Triviño A., Ramirez J.M. (2015). Ophthalmologic Psychophysical Tests Support OCT Findings in Mild Alzheimer’s Disease. J. Ophthalmol..

[46] Shi Z., Zheng H., Hu J., Jiang L., Cao X., Chen Y., Mei X., Li C., Shen Y. (2019). Retinal Nerve Fiber Layer Thinning Is Associated With Brain Atrophy: A Longitudinal Study in Nondemented Older Adults. Front. Aging Neurosci..

[47] Mishra S., Blazey T.M., Holtzman D.M., Cruchaga C., Su Y., Morris J.C., Benzinger T.L.S., Gordon B.A. (2018). Longitudinal brain imaging in preclinical Alzheimer disease: Impact of APOE ε4 genotype. Brain.

[48] Pegueroles J., Vilaplana E., Montal V., Sampedro F., Alcolea D., Carmona-Iragui M., Clarimon J., Blesa R., Lleó A., Fortea J. (2016). Longitudinal brain structural changes in preclinical Alzheimer’s disease. Alzheimer’s Dement..

[49] Fjell A.M., McEvoy L., Holland D., Dale A.M., Walhovd K.B. (2014). What is normal in normal aging? Effects of aging, amyloid and Alzheimer’s disease on the cerebral cortex and the hippocampus. Prog. Neurobiol..

[50] Bakkour A., Morris J.C., Wolk D.A., Dickerson B.C. (2013). The effects of aging and Alzheimer’s disease on cerebral cortical anatomy: Specificity and differential relationships with cognition. Neuroimage.

[51] Busche M.A., Chen X., Henning H.A., Reichwald J., Staufenbiel M., Sakmann B., Konnerth A. (2012). Critical role of soluble amyloid-β for early hippocampal hyperactivity in a mouse model of Alzheimer’s disease. Proc. Natl. Acad. Sci. USA.

[52] Ren S.-Q., Yao W., Yan J.-Z., Jin C., Yin J.-J., Yuan J., Yu S., Cheng Z. (2018). Amyloid β causes excitation/inhibition imbalance through dopamine receptor 1-dependent disruption of fast-spiking GABAergic input in anterior cingulate cortex. Sci. Rep..

[53] Ortuño-Lizarán I., Sánchez-Sáez X., Lax P., Serrano G.E., Beach T.G., Adler C.H., Cuenca N. (2020). Dopaminergic Retinal Cell Loss and Visual Dysfunction in Parkinson Disease. Ann. Neurol..

[54] Margeta M.A., Yin Z., Madore C., Pitts K.M., Letcher S.M., Tang J., Jiang S., Gauthier C.D., Silveira S.R., Schroeder C.M. (2022). Apolipoprotein E4 impairs the response of neurodegenerative retinal microglia and prevents neuronal loss in glaucoma. Immunity.

[55] Baird P.N., Richardson A.J., Robman L.D., Dimitrov P.N., Tikellis G., McCarty C.A., Guymer R.H. (2006). Apolipoprotein (APOE) gene is associated with progression of age-related macular degeneration (AMD). Hum. Mutat..

[56] Shen L., Hoffmann T.J., Melles R.B., Sakoda L.C., Kvale M.N., Banda Y., Schaefer C., Risch N., Jorgenson E. (2015). Differences in the Genetic Susceptibility to Age-Related Macular Degeneration Clinical Subtypes. Investig. Opthalmol. Vis. Sci..

