# Supplementary material for: Exploratory Longitudinal Study of Ocular Structural and Visual Functional Changes in Subjects at High Genetic Risk of Developing Alzheimer’s Disease
Source: Biomedicines. 2023 Jul 18;11(7):2024. doi: 10.3390/biomedicines11072024 (PMC10377092; doi:10.3390/biomedicines11072024)
Supplement: Supplementary file 1 [file biomedicines-11-02024-s001.zip › biomedicines-2486637-supplementary.pdf]

Supplementary Table 1. Measurements of psychophysical tests in subjects over 60 years of age

		HF- ApoE ε4- >60 yrs			HF+ ApoE ε4+ >60 yrs			p-value at 27 months HF- ApoE ε4- >60 yrs vs. HF- ApoE ε4- >60yrs
		**Baseline**	**Follow-up**	**p-valor**	**Baseline**	**Follow-up**	**p-value**	
**Visual acuity**		1.063±0.139	1.067±0.163	0.999	1.000±0.074	1.033±0.082	0.785	0.523
**Contrast sensitivity**	**3 cpd**	1.824 ± 0.179	1.830±0.122	0.257	1.686±0.166	1.782±0.134	0.180	0.613
	**6 cpd**	1.949±0.220	2.112±0.147	0.136	1.977±0.169	1.990±0.095	0.457	0.127
	**12 cpd**	1.659±0.126	1.670±0.122	0.892	1.597±0.189	1.745±0.141	0.269	0.677
	**18 cpd**	1.186± 0.126	1.300±0.155	0.096	1.158±0.296	1.250±0.190	0.461	0.309
**Colour perception**	**Total errors**	3.000(0.000-5.250)	3.000(0.000-4.500)	0.343	3.000(0.000-3.000)	3.500(0.000-5.750)	0.588	0.868
	**Tritan errors**	1.500(0.000-3.000)	0.000(0.000-1.250)	0.461	0.000(0.000-2.000)	1.000(0.000-3.250)	0.257	0.325
	**Deutan errors**	1.500(0.000-3.000)	0.000(0.000-1.250)	0.461	0.000(0.000-2.000)	0.000(0.000-4.000)	0.414	0.702
**PDT**		1.000 (0.000-1.000)	0.000(0.000-0.750)	0.705	0.500 (0.000-1.000)	0.000(0.000-1.000)	0.705	0.902
**Visual Field**	**Fixation losses**	1.000(0.000-2.000)	1.000(0.750-3.750)	0.336	0.500(0.000-1.000)	0.500(0.000-1.250)	0.317	0.204
	**False positives %**	2.000(0.000-5.000)	2.000(0.000-4.250)	0.705	0.500(0.000-2.500)	2.500(0.000-7.000)	0.109	0.681
	**%False negatives**	0.000(0.000-5.500)	2.000(0.000-10.500)	0.999	0.000(0.000-1.500)	0.000(0.000-0.000)	0.317	0.059
	**VFI (%)**	99.000(98.000-100.000)	98.500(96.750-100.000)	0.655	99.000(98.000-99.750)	99.000(98.750-100.000)	0.109	0.406
	**Mean deviation (MD)**	-0.335(-1.698-0.228)	-1.440(-2.725-0.090)	0.753	-1.440(-1.955-2.350)	-0.515(-1.443-0.230)	0.500	0.337
	**Pattern Standard Deviation (PSD)**	1.766(1.490-2.133)	2.300(1.560-5.188)	0.463	1.505(1.440-1.938)	1.640(1.435-1.845)	0.225	0.200

Median (interquartile range); *P<0.05; **P<0.01 Wilcoxon test and Mann Whitney U test.
FH; Family history; ApoE: Apolipoprotein E; cpd: cycles per degree; PDT: perception digital test; VFI: Visual Field Index; MD:mean deviation; PSD:patterns Standard Desviation

Supplementary Figure 1

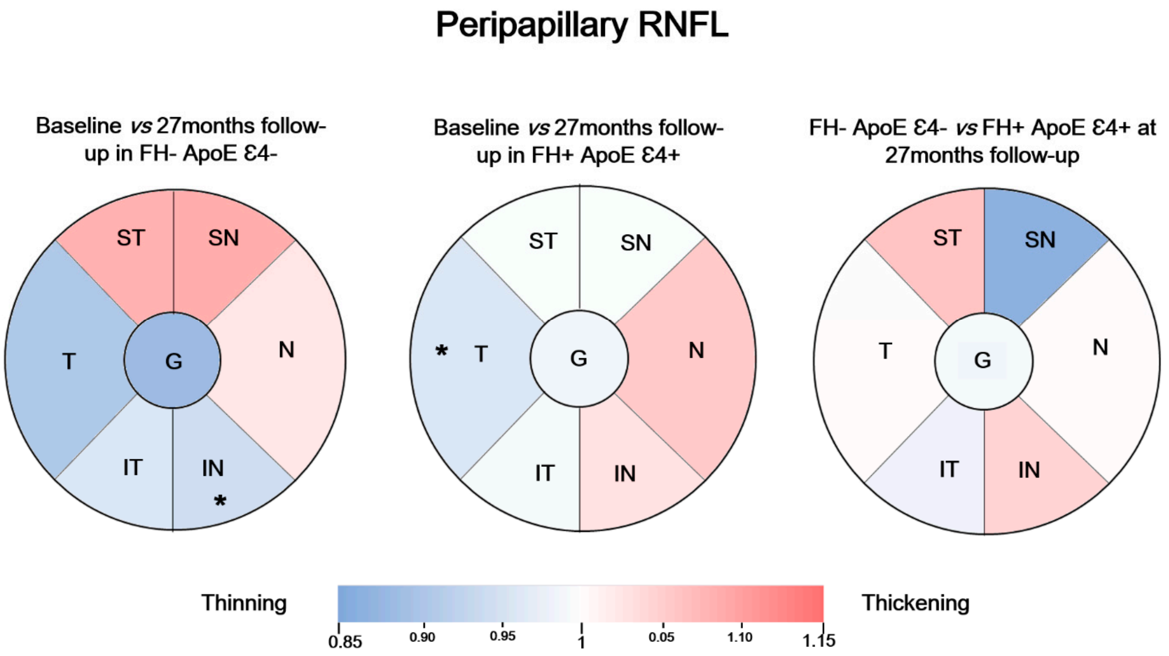

Supplementary Figure 1. Colorimetric differences in the peripapillary retinal nerve fiber layer thickness between baseline measurements and 27 months follow-up in the FH- ApoE Ɛ4- and FH+ ApoE Ɛ4+ groups and between groups at the 27 months follow-up. In red, thickening; in blue, thinning. (RNFL: retinal nerve fiber layer; ST: supero-temporal; SN: supero-nasal; N: nasal; IN: infero-nasal; IT: infero-temporal, T: temporal and G: global). *p < 0.05; **p<0.01. Wilcoxon test.
